# An experimental examination of alcohol consumption, alcohol expectancy, and self‐blame on willingness to report a hypothetical rape

**DOI:** 10.1002/ab.21745

**Published:** 2017-12-15

**Authors:** Heather D. Flowe, John Maltby

**Affiliations:** ^1^ School of Psychology University of Birmingham Birmingham UK; ^2^ School of Neuroscience, Psychology and Behavior University of Leicester Leicester UK

**Keywords:** alcohol, alcohol expectancy, rape, self‐blame, sexual assault

## Abstract

This study experimentally examined the role of victim alcohol intoxication, and self‐blame in perceiving and reporting rape to the police using a hypothetical interactive rape scenario. Participants (*N* = 79) were randomly assigned to consume alcohol (mean BAC = 0.07%) or tonic water before they engaged in the scenario. Alcohol expectancy was manipulated, and participant beliefs about the beverage they thought they had consumed and their feelings of intoxication were measured. Alcohol consumption and expectancy did not affect the likelihood that the nonconsensual intercourse depicted in the scenario was perceived and would be reported as rape. Participants with higher levels of self‐blame were less likely to say they would report the hypothetical rape. Self‐blame levels were higher for participants who believed they had consumed alcohol, and were associated with increased feelings of intoxication. The implications are discussed.

## 1 INTRODUCTION

Studies find that between 30% and 74% of sexual violence victims were alcohol‐intoxicated during the crime (e.g., Abbey, 2002; Horvarth & Brown, 2006; Koss & Dinero, 1989; Palmer, Flowe, Takarangi, & Humphries, 2013; Testa & Livingston, 2000). According to victim surveys and interviews, people who were alcohol‐intoxicated during the assault rather than sober are less likely to report rape to the police (Clay‐Warner & Burt, 2005; Finkelson & Oswalt, 1995; Resnick et al., 2000; Sawtell, 2009; Wolitzky‐Taylor et al., 2011).

Intoxicated victims may be less likely than sober victims to report because, unlike their sober counterparts, they do not perceive the incident as rape. Victim surveys (e.g., Testa & Livingston, 2000), and experimental research (Loiselle & Fuqua, 2007; c.f. Pumphrey‐Gordon & Gross, 2007) find that alcohol reduces people's ability to detect sexual assault threats and risks (e.g., the perpetrator's attempts to isolate the victim), even at low doses (i.e., BAC = 0.04%). Alcohol can also affect memory for rape. Alcohol intoxication during encoding reduces the volume, but not the accuracy, of information remembered about rape scenarios 24 hr and 4 weeks later (Flowe, Takarangi, Humphries, & Wright, 2016).

Self‐blame is common in alcohol‐involved rape, and can affect rape reporting (Ullman, 2010). There are two types of self‐blame that have been studied, behavioral and characterological (Janoff‐Bulman, 1979). With behavioral self‐blame, the cause of rape is attributed to controllable specific actions on the part of the victim (e.g., “I did not scream for help”), whereas with characterological self‐blame, the cause of rape is attributed to uncontrollable and stable factors related to oneself (e.g., “I am too trusting”). People who blame themselves for rape are less willing to disclose the attack to others, and feel greater shame with respect to themselves, their bodies and behavior (Vidal & Petrak, 2007). Further, shame has been reported as the number one barrier against reporting rape (Sable, Danis, Mauzy, & Gallagher, 2006), and victims also say they do not report because they wish to avoid further humiliation (Povey et al., 2008).

Victims’ interpretations of their experience are likely to be affected by stereotypes and cultural expectations regarding characteristics of ‘real’ rape (see Girard & Senn, 2008). These expectations include that, in real rape, the victim is not alcohol‐intoxicated, the perpetrator is someone who is a stranger to the victim, and the victim immediately reports the rape to the police (e.g., Lonsway & Fitzgerald, 1994). In line with this, sober participants tend to view a woman who is drinking alcohol instead of a non‐alcoholic beverage as more interested in having sex with a dating partner (e.g., Abbey, Zawacki, & McAuslan, 2000; George, Stoner, Norris, Lopez, & Lehman, 2000), and are more likely to hold a female victim accountable for rape if she consumed alcohol beforehand (e.g., Sims, Noel, & Maisto, 2007). Alcohol also seems to affect character evaluations, with sober participants negatively judging women who voluntary consume alcohol in risky situations (e.g., Grubb & Turner, 2012). Victims who were intoxicated during the assault can also experience negative social reactions that focus on the victim's pre‐assault drinking (Relyea & Ullman, 2015). Further, in evaluating vignettes of dating partner violence, women blame an intoxicated perpetrator less than one who was sober, and say they would be less likely to report him to the police (Katz & Arias, 2001). Further, there is evidence that legal decision makers hold complainants who were intoxicated more accountable than those who were sober (e.g., Evans & Schreiber Compo, 2010; Schuller & Stewart, 2000).

Previous research investigating the link between alcohol and victim reactions to rape has relied on asking women to retrospectively report about their past experiences. This type of research is valuable because it allows for examining the role of alcohol intoxication in the context of actual cases, and therefore, across a range of real world circumstances. This research is limited, however, because causal conclusions about the effects of alcohol on self‐blame and women's willingness to report rape cannot be drawn. The present study sought to complement this previous work by experimentally investigating the effects of alcohol and the role of self‐blame using a hypothetical scenario.

We randomly assigned women to consume alcohol or tonic water and manipulated alcohol expectancy by leading half of the participants in each beverage condition to believe that they were consuming alcohol, and the other half tonic water. Seven days later, participants were surveyed about whether they thought rape had occurred and whether they would report it to the police had it actually happened to them. Participants also completed a self‐blame measure (Frazier, 2003), and reported whether they thought they had consumed alcohol as a check on our expectancy manipulation as per recommended practice, given that expectancy manipulations can often fail to produce effects (see Testa et al., 2006). Due to funding limitations, we tested only women because rape disproportionately affects women; however, men can also be victims of rape (U.S. Department of Justice, 2010) and we will return to this point later.

Alcohol expectancy set was included as a variable in the experimental design because alcohol expectancies can bias evaluations of sexual situations. Research to date has not examined the role of alcohol expectancies in women's rape reporting. In other work, women who report more outcome alcohol expectancies (e.g., expecting to be more sexually responsive after consuming alcohol) are more likely to have a history of severe sexual victimization (Testa & Dermen, 1999), and they are less likely to indicate that they would resist in hypothetical rape scenarios (Pumphrey‐Gordon & Gross, 2007). Men who consumed or expected to consume alcohol are less likely to discriminate consensual from nonconsensual sexual behavior (Gross, Bennett, Sloan, Marx, & Juergens, 2001; Marx, Gross, & Adams, 1999 *)*. Likewise, female victims who expect to consume alcohol may also be less likely than their counterparts to perceive and report non‐consensual sexual intercourse as rape.

To summarize, we predicted on the basis of past research that women who consumed or who expected to consume alcohol would 1) be less likely to perceive the non‐consensual sexual intercourse depicted in the scenario as rape, 2) be less likely to report it to the police as rape, and 3) report higher levels of self‐blame for the attack.

## 2 METHOD

### 2.1 Participants

A sample size of 72 was needed to examine the effect alcohol consumption on rape reporting (with 36 in each beverage group), according to a power analysis (80% power, alpha = 0.05, two‐tailed) (Kadam & Bhaleraol, 2010) that used an effect size (utilized effect size *d* = −0.36, *SE *= 0.92) that was derived (see Chinn, 2000) from past research on the association between victim alcohol intoxication and rape reporting (Clay‐Warner & Burt, 2005). No previous study has examined the relationship between alcohol expectancy and rape reporting, and we had no prior reason to believe that the effect size for the expectancy manipulation would differ from the effect size for beverage. Beverage and expectancy were not predicted to interact; thus, power analyzes to test for the interaction effect were not carried out.

We recruited a total of 79 female staff and students who were between the ages of 18 and 32 years (*M* = 20.59, *SD* = 2.25 years) from the University of Leicester. This age range is ideal because sexual assault disproportionately affects young adult women (U.S. Department of Justice, 2010). The study received ethical approval from the University Ethics Committee (i.e., the IRB). Written informed consent was obtained prior to participation, and participants were told verbally and in writing that they could withdraw from the study at any time without penalty. No women withdrew from the study. Women received £4 per hr for their participation.

### 2.2 Design

A 2 beverage (consumed tonic water or alcohol) x 2 expectancy (told tonic water or alcohol) between participants design was employed. Women were randomly assigned to conditions. The outcome measures were rape perception, and rape reporting. A total of 41 women were randomly assigned to the tonic water condition (22 expected alcohol and 19 expected tonic), and 38 to the alcohol condition (20 told alcohol and 18 told tonic).

### 2.3 Materials and procedure

An advertisement for female social drinkers was circulated around campus. Prospective participants completed an online pre‐screening and were told that the study was about sexual and dating forms of behavior, and might include discussions about sexual assault. Women were invited to participate if they scored less than 11 on the Alcohol Use Disorders Identification Test (AUDIT), which assesses whether a person's drinking is harmful, hazardous, or dependent (Babor, Higgins‐Biddle, Saunders, & Monteiro, 2001). Also, women could participate if they indicated that they did not have any health problems, were not pregnant and did not use any prescription drugs that would cause an adverse reaction to alcohol. Participants were asked not to consume any alcohol or any food 4 hr prior to participation; all participants reported they had followed this instruction.

Women participated individually. On the day of the study, the experimenter confirmed the participant's answers on the AUDIT and health questionnaires. The participant then took a urine‐based pregnancy test to confirm she was not pregnant. Participants also signed a study release form, which stated that experimenters would ask that the participant remain in the laboratory until their BAC level was less than 0.02% and advised her not to drive an automobile or operate heavy machinery for the rest of the day.

The study proper then commenced. First, an AlcoHAWK portable breathalyzer was used to confirm that the participant's BAC was 0.00%. Next, the participant was provided with three red cups, which either contained an alcoholic or a tonic water beverage, depending on the condition to which the participant had been assigned. In the alcohol beverage condition, five parts tonic water to one part vodka were combined to achieve a BAC of 0.08%. The quantity of alcohol the participant was given was based on her height and weight, following the formula given in Curtin and Fairchild (2003). Note that laboratory research typically does not employ alcohol dosage levels that result in a BAC over 0.08% for ethical reasons. In the tonic water beverage condition, women were given three red cups filled with tonic water. In all beverage conditions, the cups contained vodka soaked limes and were rimmed with vodka to disguise the alcohol condition to which women had been assigned. Participants consumed each cup within 5 min.

To control alcohol expectancy, half of the participants in each beverage condition were told that they were going to consume alcohol, whereas the other half were told that they were going to consume only tonic water. Additionally, the cups were labeled with “tonic” or “vodka and tonic,” depending on the expectancy condition.

Thirty minutes later, the scenario (see Appendix A for an example of a scenario that was used) was administered. At this time, mean BAC in the tonic water group was 0.00% (*SD* = 0.00), and 0.07% (*SD *= 0.02) in the alcohol group. The scenario was administered using the *participant choice* procedure (see Flowe, Ebbesen, & Putcha‐Bhagavatula, 2007; Flowe, Stewart, Sleath, & Palmer, 2011), which allows the participant to control the activity occurring in the scenario between her and a prospective male dating partner. The basic plot of the scenario was that the participant encounters a man at a location, and soon he begins to flirt with her. The male was described as consuming alcohol, and a picture of his beverage was shown. It was important to control for perpetrator alcohol consumption because it is associated with rape disclosure (Rickert, Wiemann, & Vaughan, 2005). There were four scenario locations (i.e., bar, her house, his house, and a party) that were crossed with four different versions of the man (i.e., each version had unique biographical information about the man, such as his occupation, the type of car he drove, his hometown, his hobbies, etc.) to maintain the generalizability of the findings.

The scenario was computer‐administered. It was presented in writing on the computer screen with accompanying pictures. Participants also listened, over headphones, to a recording of the scenario text, which was read aloud by a female. The participant was told that the scenario would depict a situation between her a man. She was instructed that the scenario would unfold one stage at a time, and that at the end of each stage, she would be given a choice about whether to remain in the scenario or to end it (i.e., to tell the man that she wanted to “call it a night”). The programme that administered the survey recorded the stage at which the participant withdrew as a measure of consent level.

For as long as the participant chose to remain in the scenario, the sexual activity was described as consensual. If the participant elected to “call it a night” at any stage, a rape continuation scenario (see Appendix A for an example of a rape continuation scenario we used) was presented. If participants withdrew *after* they were already inside the house, the participant read that the man in the scenario would not take “no” for answer, and has restrained her and has had non‐consensual sexual intercourse with her. If participants withdrew before they were alone in the house, they would read that she and the man parted company, but the man later broke into her home, said he that would not take ‘no’ for an answer, restrained her, and had sexual intercourse with her against her will.

After reading the scenario, the participant remained in the lab for 2 hr if they had not consumed alcohol (to make it less obvious to them the beverage condition to which they had been assigned). Women who had consumed alcohol remained in the lab until they reached a BAC of 0.02% or lower. The research assistants stayed with the participant during this time. The participant could elect to read, browse the Internet, or watch a movie; but, more often than not, she conversed with the undergraduate and/or postgraduate research assistants, who were under strict instructions to not discuss the study with the participant. The research assistants had been trained to observe the participant for adverse reactions following the scenario. Women were told and given written information to take with them about counselling services on and off campus. They were also told that they would receive a link to an online questionnaire a week later, and that they should complete and submit the questionnaire on the day it was received

Participants completed the post‐scenario survey 7 days later, and it contained the measures of interest. Victims often do not immediately disclose rape, and among those who do report to the police, they are interviewed 14 days later on average (Westera, Kebbell, & Milne, 2011). Therefore, it seemed important that the present study capture women's perceptions of the scenario and levels of self‐blame after a delay given the generalization context. The participant was asked to indicate whether she thought the sexual intercourse described was consensual, and whether she would report it to the police as rape, using a Likert‐type scale, anchored at, 1, “Definitely No,” and 11, “Definitely Yes.” Following this, participants completed the characterological and behavioral self‐blame subscales of the Rape Attribution Questionnaire (RAQ) (Frazier, 2003). Items on the RAQ are measured on a five‐point scale that is anchored from “Never” to “Very Often.” The characterological self‐blame scale contains items that measure the belief that one has contributed to the sexual assault (e.g., “I am just the victim type”). Items from the behavioral self‐blame scale measure the belief that one's behavior led to the sexual assault (“I just put myself into a vulnerable position”). Lastly, participants were asked what drink they thought they had consumed, and to indicate how intoxicated they felt while reading the scenario using a Likert‐type scale, anchored from 0, “Completely Sober,” to 10, “Completely Intoxicated.”

Participants returned to the lab for a one‐on‐one in‐person debrief that explained the purpose of the study and queried the participant regarding their well‐being following the study. No participant withdrew from the study and no adverse events were reported.

## 3 RESULTS

### 3.1 Measures and data analysis

Categorical variables were dummy coded. For beverage, alcohol was dummy coded as “1,” and tonic as “0”. For expectancy, told alcohol was dummy coded as “1,” and told tonic as “0.” For the variable that measured whether women thought they had been given alcohol, “alcohol” responses were dummy coded as “1” and “tonic” as “0”; hereafter, this variable is termed “alcohol beliefs.” As for the RAQ, items were summed within each subscale and across subscales to form a composite self‐blame measure. Cronbach's alpha was calculated, and the results obtained for the subscales (behavioral self‐blame: *α* = 0.78; characterological self‐blame: *α* = 0.75) and for composite self‐blame (*α* = 0.87) indicated acceptable reliability levels (see DeVellis, 2003).

### 3.2 Preliminary analyzes

First we checked whether scenario version and scenario man influenced the rape perception and rape reporting dependent variables. The dependent variables were separately analyzed with a 4 scenario version x 4 scenario man between subjects ANOVAs, with the between subjects factors entered as random effects. Note that power is low for testing the scenario version x scenario man interaction effect. Rape perception scores were not significantly affected by scenario version, *F*(3, 63) = 0.14, *p* = 0.93, $\eta_{p}^{2}$ = 0.04, or by scenario man, *F*(3, 63) = 0.12, *p* = 0.95, $\eta_{p}^{2}$ = 0.04, nor did scenario version and scenario man significantly interact, *F*(9, 63) = 1.10, *p* = 0.38, $\eta_{p}^{2}$ = 0.14. Likewise, rape reporting was not significantly affected by scenario version, *F*(3, 63) = 1.51, *p* = 0.38, $\eta_{p}^{2}$ = 0.27, or by scenario man, *F*(3, 63) = 0.69, *p* = 0.58, $\eta_{p}^{2}$ = 0.18, and the interaction between scenario version and scenario man was also non‐significant, *F*(9, 63) = 0.98, *p* = 0.47, $\eta_{p}^{2}$ = 0.12. Thus, scenario version and scenario man will not be further considered.

The next analysis examined whether the expectancy and beverage manipulations affected women's feelings of intoxication as intended. A 2 (beverage) x 2 (expectancy) between subjects ANOVA indicated that women who had consumed alcohol reported feeling more intoxicated than those who had consumed tonic water (*M* = 5.51, SEM = 0.38 versus *M* = 1.47, SEM = 0.37), a significant main effect for beverage, *F*(1, 75) = 58.12, *p *< 0.0001, $\eta_{p}^{2}$ = 0.44. Women who were told alcohol reported feeling as intoxicated as those who were told tonic water (*M* = 3.63, SEM = 0.37 vs. *M* = 3.25, SEM = 0.40, respectively), *F*(1, 72) = 0.50, *p *> 0.05, $\eta_{p}^{2}$ = 008. Beverage and expectancy did not significantly interact, *F*(1, 75) = 0.63, *p* = 0.43, $\eta_{p}^{2}$ = 0.02.

The majority (61%) of women in the study thought they had consumed alcohol. We tested whether the beverage women thought they had consumed was affected by the experimental manipulations. A 2 beverage x 2 expectancy logistic regression analysis was conducted, with alcohol beliefs as the dependent variable. Women were more significantly more likely to respond that they had consumed alcohol if they actually had consumed alcohol (*b *= 3.32, SE = 1.13, *p* = 0.003) and if we told them they had consumed alcohol (*b* = 3.87, SE = 1.13, *p* < 0.001). Beverage and expectancy did not significantly interact (*b* = 16.88, SE = 8987.21, *p* = 0.99).

However, the alcohol expectancy manipulation failed for a substantial number of women (*n* = 18); 32% (12 out of 37) of women who were told they were consuming tonic thought they were given alcohol, and 14% (6 out of 42) who were told they were consuming alcohol thought they were given tonic water. These results suggest that some participants had different expectations about the beverage they were consuming than they were told. Therefore, in the analyzes that follow, the beverage that women believed they had consumed (hereafter termed “alcohol beliefs”) also served as an expectancy measure.

Overall, 83% (*n* = 66) of participants read the rape scenario continuation (i.e., they “called it a night” at some point, and therefore, read the rape depiction). In analyzing the effects of alcohol and self‐blame on rape reporting, only the participants who did not consent to sexual intercourse (i.e., those who read the rape continuation) were included in the analysis.

Table 1 presents the zero‐order correlation coefficients for the study variables. Pearson's *r* was used to analyze the associations between continuous variables, whereas Spearman's *rho* was used when one of the variables was dichotomous. The extent to which women believed the sexual intercourse to be consensual was significantly and positively associated with every self‐blame measure, but it was not significantly associated with any of the alcohol variables. Self‐blame was also positively and significantly correlated with feelings of intoxication. Rape reporting was also significantly and negatively associated with every self‐blame measure (see Table 1), and with the extent to which women believed that the sexual intercourse was consensual. Rape reporting was not associated with the beverage women consumed or expectancy.

**Table 1:** Zero‐order correlations for the study variables among participants (*N* = 66) who read the rape depiction

	1	2	3	4	5	6	7	8	9	10
1 Beverage		0.30	0.47**	0.72**	0.17	0.19	0.13	0.00	0.03	−0.17
2 Expectancy			0.52**	0.07	0.00	−0.08	0.15	−0.01	0.00	−0.12
3 Alcohol beliefs				0.59**	0.26*	0.19	0.34**	0.02	0.03	−0.28*
4 Feelings of intoxication					0.29*	0.27*	0.26*	−0.03	0.05	−0.11
5 Composite self‐blame						0.95**	0.87**	0.18	0.20	−0.31
6 Characterological self‐blame							0.67**	0.17	0.21	−0.28*
7 Behavioral self‐blame								0.14	0.14	−0.30*
8 Consent level									0.16	−0.09
9 Rape consensual										−0.45**
10 Rape reporting										

*p *< 0.05, two‐tailed

*p* < 0.001, two‐tailed

In what follows, the rape perception and rape reporting results with beverage and expectancy included in the model are presented first. The rape perception and rape reporting results with beverage and the beverage that women thought they had consumed (referred to herein as “alcohol beliefs”) included in the model are reported next.

### 3.3 Rape perceptions

To assess the effects of self‐blame and alcohol on whether women believed the rape scenario depicted consensual intercourse, a linear regression analysis was carried out including beverage, expectancy, and composite self‐blame as predictors. Composite self‐blame was included in the model because characterological and behavioral self‐blame were highly correlated. Both types of self‐blame were associated with rape perception and reporting to the same extent (see Table 1). Bootstrapping (*n* = 1,000 samples) was employed to derive estimates of the regression estimates and confidence intervals and to test the statistical significance of the coefficients (Efron & Tibshirani, 1993). The overall model was not significant, *F*(3, 62) = 0.97, MSE = 4.83, *p* = 0.41, *R* ^2^_adjusted_ = 0.00. None of the predictors were statistically significant (beverage: *b* = 0.08, SE = 0.54; *t*(62) = 0.15, *p* = 0.88; expectancy: *b* = 0.27, SE = 0.55; *t*(62) = 0.48; composite self‐blame: *b* = 0.06, SE = 0.03, *t*(62) = 1.57, *p* = 0.12). Adding the alcohol x expectancy interaction term to the model did not improve model fit, *R* ^2^_change_ = 0.014, *F*_change_ (1, 61) = 2.38, *p* = 0.12, and the resulting overall model was not statistically significant, *F*(4, 61) = 1.34, *p* = 0.26, *R* ^2^_adjusted_ = 0.02, and nor were any of the coefficients (*p*s > 0.12).

Another linear regression analysis was carried out, including beverage, alcohol beliefs, and composite self‐blame as predictors. The overall model was not significant, *F*(3, 62) = 0.89, MSE = 4.44, *p* = 0.45, *R* ^2^_adjusted_ = 0.00 and none of the predictors were significant (beverage: *b* = 0.10, SE = 0.62; *t*(62) = 0.16, *p* = 0.87; alcohol beliefs: *b* = −0.02, SE = 0.64; *t*(62) = 0.97; composite self‐blame: *b* = 0.06, SE = 0.03, *t*(62) = 1.57, *p* = 0.15). Adding the alcohol x expectancy interaction term to the model did not improve model fit, *R* ^2^_change_ = 0.014, *F*_change_ (1, 61) = 0.91, *p* = 0.34, and the overall model was not significant (*F*(4, 61) = 0.89, *p* = 0.47, *R* ^2^_adjusted_ =0.00) nor were any of the coefficients (*p*s > 0.11).

### 3.4 Rape reporting

To assess the relationship between alcohol and composite self‐blame on rape reporting, a linear regression analysis was conducted, including beverage, expectancy, and composite self‐blame as the predictors. Bootstrapping (*n* = 1,000 samples) was employed in the analysis. The overall model was statistically significant, *F*(3, 62) = 2.76, *p* = 0.049, MSE = 11.22, *R* ^2^_adjusted_ = 0.07. Composite self‐blame was significantly and negatively associated with rape reporting (*b* = −0.08, SE* *= 0.03, CI_0.95_: −0.14 to −0.02, *t*(62) = −2.48, *p* = 0.02). However, beverage (*b* = −0.33, SE = 0.47, *p* = 0.50) and expectancy (*b* = −0.47, SE = 0.51, *p* = 0.38) were not significant predictors of rape reporting. The fit of the model was not improved by adding the interaction term for beverage and expectancy (*R* ^2^_change_ = 0.004, *F_change_* (1, 61) = 0.24, *p* = 0.62), and the overall model was not significant (*F*(4, 61) = 2.11, *p* = 0.09, *R* ^2^_adjusted_ = 0.06), nor were any of the predictors (*p*s < 0.62) except for composite self‐blame (*b* = −0.08, SE* *= 0.03, CI_0.95_: −0.14 to −0.01, *t*(61) = −2.45, *p* = 0.02).

A second linear regression analysis was conducted to analyze rape reporting, with beverage, alcohol beliefs, and composite self‐blame as predictors. The overall model was marginally statistically significant, *F*(3, 62) = 2.72, *p* = 0.05, MSE = 11.08, *R* ^2^_adjusted_ = 0.07. Composite self‐blame was significantly and negatively associated with rape reporting (*b* = −0.07, SE* *= 0.03, CI_0.95_: −0.13, −0.006, *t*(62) = −2.20, *p* = 0.03). However, beverage (*b* = −0.12, SE = 0.56, *p* = 0.84) and alcohol beliefs (*b* = −0.51, SE = 0.55, *p* = 0.36) were not significant predictors of rape reporting. The fit of the model was not improved by adding the interaction term for beverage and alcohol beliefs (*R* ^2^_change_ = 0.001, *F_change_* (1, 61) = 0.08, *p* = 0.77), and the overall model was not significant (*F*(4, 61) = 2.03, *p* = 0.10, *R* ^2^_adjusted_ = 0.06), nor were any of the predictors (*p*s < 0.60) except for composite self‐blame (*b* = −0.08, SE* *= 0.03, *CI*_.95_: −0.07 to −0.03, *t*(61) = −2.20, *p* = 0.03).

### 3.5 Self‐blame

Given the relationship between composite self‐blame and rape reporting, we modeled self‐blame as a function of beverage and expectancy, and also as a function of beverage and alcohol beliefs. Bootstrapping (*n* = 1,000 samples) was employed in the analysis. When composite self‐blame was modeled using beverage and expectancy as predictors, the overall model was not statistically significant (*F*(2, 65) = 0.98, *p *= 0.38, *R* ^2^_adjusted_ = 0.00, nor were any of the predictors (*p*s > 0.16).

When composite self‐blame was analyzed using beverage and alcohol beliefs as predictors, the overall model was marginally significant, *F*(2, 63) = 2.88, *p* = 0.06, MSE = 176.95, *R* ^2^_adjusted_ = 0.06. Alcohol beliefs were positively and significantly associated with composite self‐blame, *b* = 4.21, SE = 2.10, CI_0.95_: 0.017–8.28, *t*(63) = 1.92, *p* = 0.047). Beverage was not a significant predictor, *b* = 0.83, SE = 2.16, *t*(63) = 0.38, *p* = 0.69. The addition of the interaction term for beverage and alcohol beliefs did not improve model fit (*R* ^2^_change_ = 0.006, change *F*(1, 62) = 0.40, *p* = 0.53) nor was the overall model significant (*F*(3, 62) = 2.03, *p* = 0.12, *R* ^2^_adjusted _= 0.05).

## 4 DISCUSSION

When considered together in the same model, only self‐blame significantly predicted rape reporting, whereas alcohol consumption, expectancy and alcohol beliefs did not. Further, women who believed that they had consumed alcohol rather than to tonic water blamed themselves more for the rape, and women were more likely to blame themselves for the assault the more intoxicated they felt. Therefore, the results suggest alcohol consumption contributes to self‐blame in rape, and women who blame themselves may not be as likely to report. These findings will now be discussed.

It is concerning that women in the current study were more likely to blame the hypothetical rape on their behavior and character if they believed that they had consumed alcohol. Participants were not given a choice about whether they were given alcohol or not. All women were told verbally and in writing, as per the consent form, that they would be randomly assigned to consume either an alcoholic or a placebo beverage. Consequently, they should have perceived their alcohol consumption as being outside of their control. Nevertheless, women's beliefs about whether they had consumed alcohol influenced how blameworthy they felt with respect to rape that took place in the scenario. This suggests beliefs about alcohol consumption and women's accountability for rape are important factors in a victim's decision to report rape, and these beliefs can override factual information. Indeed, self‐report data indicate that victims are less likely to report rape to the police if drugs and/or alcohol facilitate it rather than force (Wolitzky‐Taylor et al., 2011). Further, mock jurors condemn intoxicated complainants, even when intoxication was involuntary (Finch & Munro, 2005). In actual cases, particularly where victims have control over their alcohol consumption, the association between voluntary alcohol consumption and self‐blame could possibly be even stronger than we observed in this experiment.

The beverage women consumed did not affect their reactions to rape. Alcohol directs attention to the most immediate and salient cues in the environment, resulting in peripheral and relatively weak cues being given less attention (Steele & Josephs, 1990). In the present study, the alcohol‐related shift in attention allocation may have allowed intoxicated women to just as accurately perceive rape as sober women because their attention was directed toward the behavior of the perpetrator, who was the most immediate and central character in the scenario. Research has found that both sober and intoxicated women attend to a greater extent to the perpetrator in a rape scenario than more peripheral aspects, such as bystanders (Flowe et al., 2016).

The present study also contributes to the literature on the effects of alcohol consumption and expectancy on risk detection in sexual assault. Women's ability to detect sexual assault threats and risks can be minimized by alcohol consumption and expectancy (Loiselle & Fuqua, 2007, c.f. Pumphrey‐Gordon & Gross, 2007). The present study did not evaluate women's ability to detect risks and threats, but instead focused on the effects of alcohol consumption and expectancy on women's interpretation and reporting of hypothetical rape. Considering the literature as a whole, alcohol consumption and expectancy seem to compromise a victim's ability accurately and rapidly detect sexual assault risks. Once an assault has occurred, our results suggest that intoxicated victims are just as able as their sober counterparts to accurately perceive non‐consensual sexual intercourse as rape. This suggests that although intoxicated victims accurately perceive rape and remember it as such, they are less willing than sober victims to report it to legal authorities. Further, self‐blame was associated with women's perceptions of whether they had consumed alcohol and their feelings of intoxication, not their actual alcohol consumption. This suggests that alcohol expectancies, not alcohol's physiological effects, influence women's reactions to rape.

Self‐blame predicted rape reporting even after controlling for alcohol consumption and expectancy. This indicates that the effect of self‐blame on rape reporting is also caused by other factors besides the victim having had alcohol. There are a number of variables in addition to victim alcohol use (e.g., Cameron & Stritzke, 2003) that increase victim blame for rape; they include victim attire (e.g., Abbey, Cozzarelli, McLaughlin, & Harnish, 1987) and victim gender role conformity (e.g., Grubb & Turner, 2012), and these factors may also affect victim self‐blame. Further, a number of variables that tend to co‐occur with alcohol that may also impact self‐blame and perceptions of accountability, including victims attending drinking establishments, agreeing to be alone with the assailant, and/or consenting to some sexual activity. These factors were also present in our scenarios. Research is needed to untangle this constellation of alcohol‐related factors to determine how they affect attributions of responsibility for rape. Alongside this, we need to study how negative social reactions can be effectively challenged to increase rape reporting.

The results also have implications for models of victim self‐blaming. In the aftermath of rape, victims frequently blame themselves. Higher levels of self‐blame have been linked to greater psychological distress and greater risk of revictimization (e.g., Miller, Markman, & Handley, 2007). On the one hand, it has been argued that behavioral self‐blame is an adaptive response following rape, as it allows the victim to regain a sense of control and cope (Janoff‐Bulman, 1979). Intoxicated victims may identify their voluntary alcohol consumption as the cause of the rape, and as something that they can modify and control to prevent rape from occurring in the future. Further, engaging in behavioral rather than characterological self‐blame has been associated with better coping (Breitenbecher, 2006; Hill & Zautra, 1989; Koss, Figueredo, & Prince, 2002; Ullman, Townsend, Filipas, & Starzynski, 2007). On the other hand, poorer coping post‐rape has been reported in victims who engage in self‐blame, regardless of whether it is characterological or behavioral (e.g., Frazier, 1990; Meyer & Taylor, 1986). Further, victims who self‐blame may attribute rape to behavioral as well as characterological factors, rather than just one or the other (Frazier, 1990). Our results are consistent with this. We found behavioral and characterological self‐blame were positively associated. Victims who attribute rape to their alcohol consumption behavior may find it difficult not to also implicate their character. Given the prevalence of victim intoxication in rape cases, and that self‐blame is associated with poorer coping and revictimization, further work is needed to understand the attribution process and psychological outcomes for victims who were alcohol‐intoxicated during rape. This work should also consider the impact of both negative and positive social reactions to alcohol‐involved rape (see Lorenz & Ullman, 2016) when victims disclose informally (e.g., to friends and relatives) and formally (e.g., police, medical professionals), and how initial reactions affect subsequent disclosures or lack thereof.

The results could be used to develop educational programmes about the role of alcohol in rape victim self‐blame. Training programmes about self‐blame seem particularly important for first responders (e.g., police, medical and mental health professionals), who are likely to shape the victims’ perceptions of self‐blame and whether they should pursue legal remedy. Research is also needed to understand how first responders and others can best support victims who disclose. Further, our results imply that attributional retraining (see Murdock & Altmaier, 1991) as a part of treatment and recovery programmes may be important for victims who were alcohol‐intoxicated during rape.

One limitation of the current study was that specific alcohol expectancies (see Testa & Dermen, 1999) were not measured. Hence, the role that specific types of outcome expectancies play in rape attributions and rape reporting is an outstanding research question. Future work could also directly examine possible reasons why victims who were alcohol‐intoxicated during rape are less willing to report, and specifically explore the role of victim alcohol intoxication behavior pre‐ and post‐assault in rape attributions (which the RAQ does not measure). Further, the BAC levels investigated (mean BAC = 0.07%) may limit the extent to which the present results can be generalized. BAC levels in actual cases have been found to range from 0.04% to 0.39% (mean BAC = 0.19%) (Hagemann et al., 2013). Thus, more field research with actual victims is needed to understand how higher levels of intoxication affect rape perceptions and legal reporting.

Our conclusions should be viewed with some caution because of how women respond to a hypothetical situation in a laboratory may differ from how they would react if the situation occurred in real life. Having said this, the effects of alcohol on rape reporting in the real world might be even stronger than that found in the present research given the intense levels of scrutiny that victims are under in real world cases. Lastly, there is little known about the role of self‐blame in men's construal and reporting of rape. We have no reason to think that our results would not apply to men, but this is an empirical question that needs to be addressed.

In sum, participants who believed that they had consumed alcohol rather than a non‐alcoholic beverage engaged in more self‐blame. Participants who reported higher levels of self‐blame indicated that they would be less willing to report the hypothetical rape to the police. Further research is needed to better understand the role of alcohol in how victims attribute responsibility for rape, and the implications this has for rape prosecution and victim recovery.

## CONFLICTS OF INTEREST

The author has no conflicts of interest in relation to the research reported in this manuscript.

## APPENDIX 1

One of the bar scenarios (with the stages labeled)* employed in the present study.

Stage 1: You decide to go to vodka revolutions bar with some of your friends. While there you see the guy pictured below, sitting with a few friends. He is wearing a smart brown top and a pair of black jeans. You notice that he's quite tall, about 6′2.” When he catches you staring, he smiles. About half an hour later, when Bruno Mars’ latest song comes on, he walks casually toward you and introduces himself. His name is Michael Davies, he's 25 years old and he seems talkative. He says he wants to use the bathroom and that he'll catch you later. A little later, you go to the old oak bar to get a drink. It is quite busy, and Michael is in front of you in the queue. He offers to buy you a drink. Michael says that he loves living in Leicester and asks whether you live nearby. Michael buys himself a beer, and passes you your beverage. You and Michael carry on chatting for a while. He asks you what you do for a living, and tells you that he is a data communications analyst. He says that he thinks you look stunning. He asks if you want to come and sit down with him for a while. You find a quiet area with a red sofa where you can sit together. Michael comments on the unusual glass lamp beside you. You talk for about 3 hr. Michael asks you whether you have any hobbies, and he tells you that he's really into surfing. He asks you what kind of films you like and says he loves comedies. He suggests maybe you should go out to the cinema with him sometime. He tells you that you are a very exciting person to be around and he'd like to get to know you more. Time flies by, and you realize it is 2.00 am and the bar is closing. You look around, but you cannot find the friends you came with. Stage 2: Michael says he cannot find his friends either, and offers to take you home. Outside it is raining. On the way to his car he puts his arm around you. Stage 3: You get inside his car, a silver ford focus, and he asks you how to get to your house. Stage 4: At your door, he leans in forward to try to kiss you. Stage 5: He asks you if he can come in and use your phone. His apple iphone is out of battery and he needs to call his roommate. Stage 6: After using your phone, he sits down on the couch. Stage 7: He tells you to come and sit beside him. Stage 8: He kisses you again. Stage 9: He whispers he's wanted to do that all evening. Stage 10: His hands begin to wander, and start caressing your back. Stage 11: He tells you that you are very sexy. Stage 12: He kisses you and strokes your stomach. Stage 13: While kissing your neck, his hands wander up your chest. Stage 14: He is rubbing against you and it is obvious that he is aroused. Stage 15: He says that he is getting aroused just looking at you and that he wants you. Stage 16: He starts to take off your clothes. Stage 17: He kisses you again and slides his hand down your underwear. Stage 18: While kissing you, he begins to touch you intimately. Stage 19: He tangles your hair up in his hands and he pulls your underwear off. Stage 20: He kisses you all over and takes his underwear off. Stage 21: He says that he will stop if you do not want to continue, because in the short time that he has known you, he has come to care for you deeply. Stage 22: He takes a condom out from his pocket. Stage 23: He pushes you down on the couch and gets on top of you. Stage 24: You have sexual intercourse.

Rape continuation scenario for participants who “called it a night”: Michael looks angry. He says that you were leading him on and tells you that you cannot say no to him now. He pushes you down onto the floor. He says that it's too hard for him to stop. He cannot resist you. He says no one will hear you if you struggle. He is on top of you and his shoulders are holding you down. You have sexual intercourse.
